# The mechanism of acute fasting‐induced antidepressant‐like effects in mice

**DOI:** 10.1111/jcmm.13310

**Published:** 2017-08-07

**Authors:** Ranji Cui, Jie Fan, Tongtong Ge, Linda Tang, Bingjin Li

**Affiliations:** ^1^ Jilin Provincial Key Laboratory on Molecular and Chemical Genetic The Second Hospital of Jilin University Changchun China

**Keywords:** BDNF, c‐Fos, fasting, 5‐HT, Depression

## Abstract

Acute fasting induced antidepressant‐like effects. However, the exact brain region and mechanism of these actions are still largely unknown. Therefore, in this study the antidepressant‐like effects of acute fasting on c‐Fos expression and BDNF levels were investigated. Consistent with our previous findings, immobility time was remarkably shortened by 9 hrs fasting in the forced swimming test. Furthermore, these antidepressant‐like effects of 9 fasting were inhibited by a 5‐HT_2A/2C_ receptor agonist (±)‐1‐(2, 5‐dimethoxy‐4‐iodophenyl)‐2‐aminopropane hydrochloride (DOI), and the effect of DOI was blocked by pretreatment with a selective 5‐HT_2A_ receptor antagonist ketanserin. Immunohistochemical study has shown that c‐Fos level was significantly increased by 9 hrs fasting in prefrontal cortex but not hippocampus and habenular. Fasting‐induced c‐Fos expression was further enhanced by DOI in prefrontal cortex, and these enhancements were inhibited by ketanserin. The increased BDNF levels by fasting were markedly inhibited by DOI in frontal cortex and hippocampus, and these effects of DOI on BDNF levels were also blocked by ketanserin. These findings suggest that the antidepressant‐like effects of acute fasting may be exerted *via* 5‐HT_2A_ receptor and particularly sensitive to neural activity in the prefrontal cortex. Furthermore, these antidepressant‐like effects are also mediated by CREB and BDNF pathway in hippocampus and frontal cortex. Therefore, fasting may be potentially helpful against depression.

## Introduction

Recently, caloric restriction or fasting has been shown to be associated with significantly improvements in brain function 1, 2, 3, 4. For example, intermittent fasting resulted in beneficial effects in reducing and severity of some neurological disorders 1, 2. Clinical evidence has also shown that fasting and caloric restriction dietary regime was effective in improving mood states, such as confusion, anger and tension 2. Other and our studies have shown that caloric restriction and fasting may improve depression in rodents 3, 4. Lutter *et al*. reported that chronic caloric restriction also caused a significant antidepressant‐like response in animal models of depression 3. Recently, our study has shown that immobility time in the forced swimming test (FST) was remarkably inhibited by 9 hrs but not 3 and 18 hrs of fasting without change of locomotor activity 4. Moreover, the antidepressant‐like actions of 9 hrs fasting were inhibited by a 5‐HT_2A/2C_ receptor agonist (±)‐1‐(2, 5‐dimethoxy‐4‐iodophenyl)‐2‐aminopropane hydrochloride (DOI). These data reveal that 5‐HT_2_ receptor may be involved in the antidepressant‐like effects induced by acute fasting.

The c‐Fos was wieldy used as a neuronal activity marker 5, 6, 7, 8, 9. Previously studies have reported that c‐Fos expression was induced by fasting in several brain regions, such as paraventricular nucleus 7, arcuate nucleus of hypothalamus 8 and amygdala 9. It is unclear, however, which specific brain regions are involved in the antidepressant‐like actions induced by acute fasting. Therefore, the effects of acute fasting, inducing antidepressant‐like effects, on c‐Fos expression were investigated in the several brain regions, particularly hippocampus, prefrontal cortex and habenular. These brain regions have been studied extensively as the potential targets of depression treatment 4, 10, 11. These data would provide the structural basis on the mechanism of antidepressant‐like effects induced by acute fasting.

In addition, cAMP response element binding protein (CREB) and brain‐derived neurotrophic factor (BDNF) pathways are widely investigated in depression 4, 10, 12, 13, 14. We have reported that the p‐CREB/CREB ratio was increased by 9 hrs fasting in the hippocampus and frontal cortex. Furthermore, 9 hrs fasting with imipramine exerted the additive antidepressant‐like effects in the FST and enhanced the p‐CREB/CREB ratio 4. In addition to CREB, BDNF is another important target of antidepressant 10, 15. Preclinical and clinical studies have shown that depression has been strongly and consistently linked to low levels of BDNF 10, 15, 16, 17. Duan *et al*. reported that caloric restriction increased BDNF levels in the striatum and cortex 18. Another interesting study showed that food restriction decreased BDNF level in hindbrain and hypothalamus, while enhanced expression in hippocampus and cortex 19. In addition, intermittent fasting normalized BDNF levels while regular feeding kept them low 20. Taken together, these data suggest that BDNF signalling may also mediate beneficial effects of fasting on antidepressant‐like effects. However, whether BDNF is involved in acute fasting‐induced antidepressant effect require further investigation.

Therefore, in this study, the effects of acute fasting, inducing antidepressant‐like effects, on c‐Fos and BDNF levels were examined.

## Materials and methods

### Animals

ICR mice (weighing 23 ± 2 g: 6‐weeks old; Jilin University, Changchun, China) were housed in standard mouse cages under a 12/12‐hrs light/dark cycle (lights on from 06:00 to 18:00 hours) in a humidity‐ and temperature‐controlled room (50 ± 10%, 23 ± 2°C). Food and water were available *ad libitum*. All mice were acclimatized to the being handled (gentled) to reduce stress before the experiments.

### Drugs

The following drugs were used: ketanserin tartrate, DOI and imipramine hydrochloride (Sigma‐Aldrich, St. Louis, MO, USA). The drugs were made with distilled water, which is also the vehicle. All the drugs were given in a constant volume of 10 ml/kg (body weight). The drug doses were according to our previous studies 4, 15.

### Forced swim test

In first FST, all mice were divided into four groups: control group received vehicle injections, 9‐hrs fasting group (from 0:00 a.m to 9:00 a.m), 18‐hrs fasting group (from 3:00 p.m to 9:00 a.m) and imipramine‐treated group (positive control group). In second behaviour test, all mice were divided into five groups: control group; 9‐hrs fasting group; 9 hrs fasting + DOI group; 9 hrs fasting + DOI + ketanserin group; imipramine group. Ketanserin (5 mg/kg, i.p.) and DOI (5 mg/kg, i.p.) were administered 30 and 5 min. before FST, respectively. In addition, in the positive control group imipramine (30 mg/kg, i.p.) was given 30 min. before FST.

The FST was essentially was reported by our studies 4, 10. In this experiment, mice were placed in a clear Plexiglas cylinder (11 cm in diameter by 25 cm height containing 20 cm of water at 25°C). All mice were then removed and allowed to dry in a separate cage before returning to their home cages. FST was recorded for 6 min. The last 4 min of FST was measured by observer blind to the experimental condition 4, 10.

### C‐Fos immunohistochemistry

C‐Fos immunohistochemistry was performed as reported by our group 5, 6. Briefly, all mice were first deeply anesthetized with chloral hydrate (100 mg/kg, i.p.), and brain perfusion with ice‐cold PBS, followed by 4% paraformaldehyde in PBS was performed. After removal, brains were post‐fixed with 30% sucrose. Serial coronal sections (30 μm thick) from different treatment groups were processed in parallel to minimize variation in the immunohistochemical labelling. Free floating sections incubated using 0.6% solution of hydrogen peroxide containing PBS. After rinsing again with PBS buffer, the sections incubated with rabbit polyclonal c‐Fos antibody (1:1000; Santa Cruz Biotechnology, Inc., Santa Cruz, CA, USA, #sc‐52) solution containing 0.3% Triton X‐100, 0.05% sodium azide and 2% normal goat serum for 72 hrs at 4°C. The sections were then washed and incubated using a secondary antibody [1:400, biotinylated goat anti‐rabbit IgG (Vector Laboratories, Burlingame, CA, USA)] at dilution in PBS buffer containing 0.3% Triton X‐100 for 75 min. at room temperature. After rinsing in PBS buffer, the sections were replaced by PBS buffer containing 0.4% avidin‐biotinylated horseradish peroxidase complex (Vector Laboratories) for another 75 min. Following washes in PBS buffer and 0.2 M sodium acetate solution, pH 6.0, the reaction was continued using the glucose oxidase–diaminobenzidine–nickel method. The sections were finally washed by 0.2 M sodium acetate solution. Thereafter, the sections were moved to chrome‐alum‐gelatine‐coated slides. We counterstained air‐dry the section with neutral red, dehydrated using a graded alcohol series, cleared in xylene and cover slipped. Bilateral c‐Fos counting was conducted on a minimum of three representative sections per level in a blind fashion. The positive cells were counted under ×200 magnification from the hippocampus and prefrontal cortex. Using light microscopy, c‐Fos‐positive neurons were identified by dense brown nuclear staining and captured with a Nikon digital camera (Eclipse 50i Microsope: Nikon, Japan). The counting c‐Fos was blind to experimental conditions. Brain regions were confirmed using the mouse brain atlas. These counts were averaged for five to eight sections in each region for each animal.

### Measurement of BDNF levels by ELISA

Enzyme‐linked immunosorbent assay (ELISA) was performed with the BDNF Emax ImmunoAssay System Kit (Promega Inc., Madison, WI, USA) according to the manufacturer's instructions 10, 15. ELISA plates (96 well; NUNC MaxiSorp, eBioscience, San Diego, CA, USA) were coated with 100 μl/well of anti‐BDNF monoclonal antibody (mAb) and incubated overnight (4°C). The incubation of plates was performed in a block and sample buffer (room temperature, 1 hr). We added samples to the coated wells (100 μl) and shook it for 2 hrs. The antigen was incubated (anti‐Human BDNF polyclonal antibody, 2 hrs, room temperature) with shaking and then incubated with an anti‐IgY antibody conjugated to horseradish peroxidase (1 hr, room temperature). We incubated plates for 15 min. (tetramethylbenzidine solution) and added 1 M hydrochloric acid (HCL) to the wells. The reaction products determined by the colorimetric were calculated (450 nm). The standard curves from each plate were plotted. BDNF level was calculated from the regression line for the BDNF standard (ranging from 7.8 to 500 pg/ml purified mouse BDNF). Sections values were above 16 pg/ml for each plate. This experiment method was reported by our previous studies 10, 15.

### Statistical analyses

All data are expressed as the mean ± S.E.M. We analysed the data using a one‐way analysis of variance (anova). When significant differences were observed, *post hoc* comparisons within logical sets of means were carried out using Tukey's test. A *P*‐value of 0.05 is considered on the borderline of statistical significance.

## Results

### 9 hrs fasting‐induced antidepressant‐like effects

**Figure 1:**
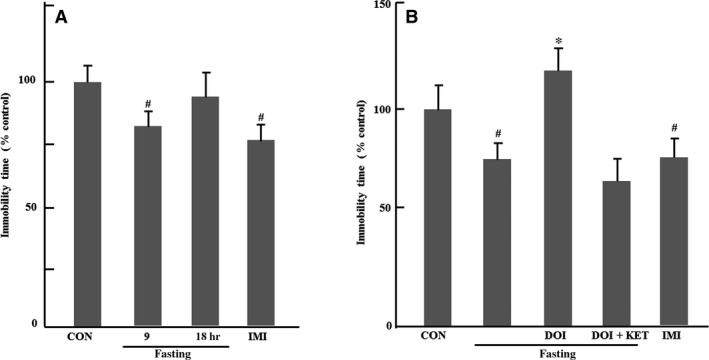
Effects of acute fasting on immobility time, normalize to control, in forced swimming test. (**A**) Effects of 9 and 18 hrs fasting on immobility time (*n* = 7–8); (**B**) Effects of 9 hrs fasting and DOI on immobility time (*n* = 8). Columns represent mean ± S.E.M. ^#^ *P *<* *0.05 as compared with control group (non‐fasting); *P <* *0.05 as compared with fasting control group; CON: control; DOI: (±)‐1‐(2, 5‐dimethoxy‐4‐iodophenyl)‐2‐aminopropane hydrochloride, 5 mg/kg, i.p.; IMI: imipramine, 30 mg/kg, i.p.; KET: ketanserin tartrate, 5 mg/kg, i.p.

As shown in Figure 1A, immobility time was remarkably inhibited by 9 hrs fasting or imipramine but not 18 hrs fasting compared to control group (9 hrs fasting: *P *<* *0.05 and imipramine: *P *<* *0.05). Furthermore, the inhibitory effect of 9 hrs on immobility time was blocked by DOI compared to fasting control group (*P *<* *0.05), and the effects of DOI were also reversed by ketanserin (Fig. 1B).

### C‐Fos in habenular

**Figure 2:**
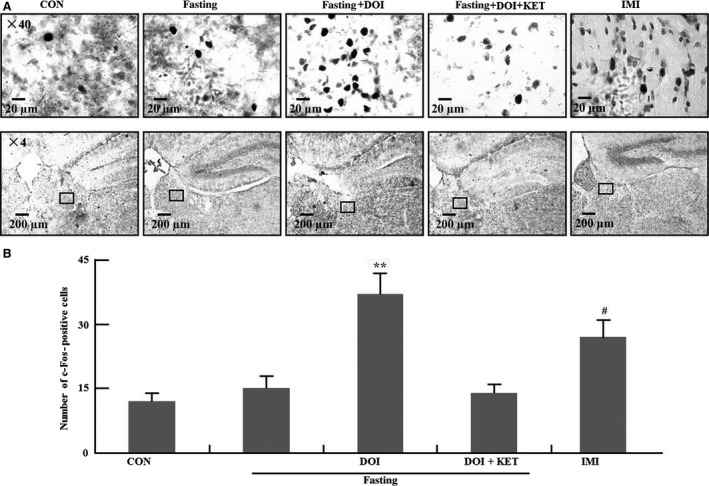
C‐Fos expression in habenular region. (**A**) Representative photomicrographs of c‐Fos staining in habenular; (**B**) Quantification of c‐Fos expression in five experimental groups. Columns represent mean ± S.E.M. (*n* = 8). ^#^ *P *<* *0.05 as compared with control group (non‐fasting); **P <* *0.01 as compared with fasting control group; CON: control; DOI: (±)‐1‐(2, 5‐dimethoxy‐4‐iodophenyl)‐2‐aminopropane hydrochloride, 5 mg/kg, i.p.; IMI: imipramine, 30 mg/kg, i.p.; KET: ketanserin tartrate, 5 mg/kg, i.p.

C‐Fos expression was examined in habenular in Figure 2. C‐Fos expression was significantly enhanced by imipramine but not 9 hrs fasting in habenular compared to control group (*P *<* *0.05). In addition, DOI also markedly increases c‐Fos expression in habenular of fasting mice (*P *<* *0.01).

### C‐Fos in prefrontal cortex

**Figure 3:**
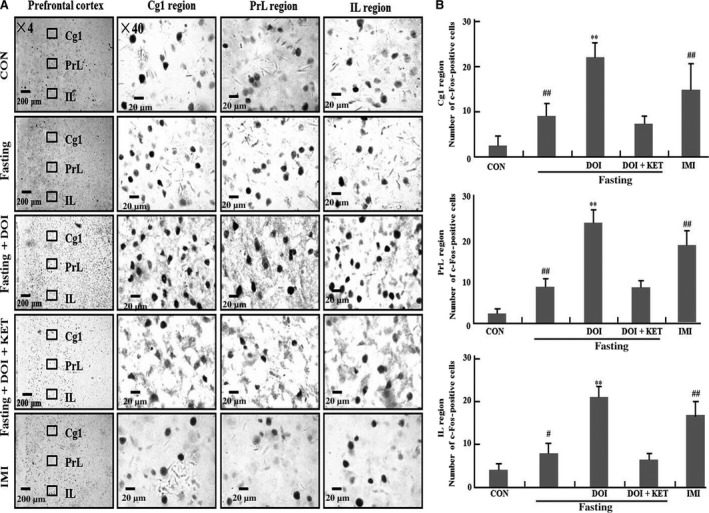
C‐Fos expression in Cg1,PrL and IL region of prefrontal cortex. Cg1: (**A**); PrL: (**B**); IL: (**C**). (**A**) Representative photomicrographs of c‐Fos staining; (**B**) Quantification of c‐Fos expression in five experimental groups.^#^ *P *<* *0.05; ^##^ *P *<* *0.01 as compared with control group (non‐fasting); *P <* *0.05; **P <* *0.01 as compared with fasting control group; CON: control; DOI: (±)‐1‐(2, 5‐dimethoxy‐4‐iodophenyl)‐2‐aminopropane hydrochloride, 5 mg/kg, i.p.; IMI: imipramine, 30 mg/kg, i.p.; KET: ketanserin tartrate, 5 mg/kg, i.p.

In Figure 3A and B, c‐Fos expression was summarized in Cg1, PrL and IL of prefrontal cortex. Nine hours fasting significantly enhanced c‐Fos expression in Cg1, PrL and IL of prefrontal cortex compared to control group (Cg1: *P *<* *0.01; PrL: *P *<* *0.01; IL: *P *<* *0.05). Furthermore, these enhancements of c‐Fos expression were further increased by DOI in fasting mice (Cg1: *P *<* *0.01; PrL: *P *<* *0.01; IL: *P *<* *0.01), and these effects of DOI was also blocked by ketanserin. Additionally, c‐Fos level was also increased by imipramine in Cg1, PrL and IL of prefrontal cortex compared to control group (Cg1, PrL and IL: *P *<* *0.01).

### C‐Fos in hippocampus

**Figure 4:**
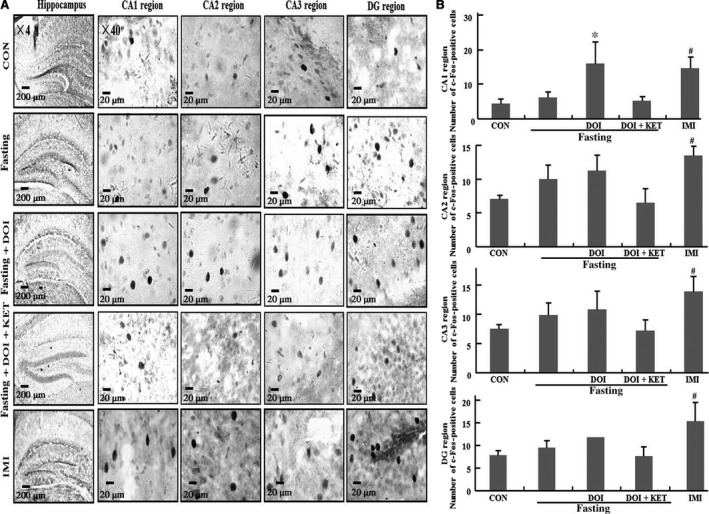
C‐Fos expression in CA1, CA2, CA3 and dentate gyrus region of hippocampus. (**A**) Representative photomicrographs of c‐Fos staining;(**B**) Quantification of c‐Fos expression in five experimental groups. Columns represent mean ± S.E.M. (*n* = 8).^ #^ *P *<* *0.05 as compared with control group (non‐fasting); *P <* *0.05 as compared with fasting control group; CON: control; DOI: (±)‐1‐(2, 5‐dimethoxy‐4‐iodophenyl)‐2‐aminopropane hydrochloride, 5 mg/kg, i.p.; IMI: imipramine, 30 mg/kg, i.p.; KET: ketanserin tartrate, 5 mg/kg, i.p.

Data of c‐Fos was shown in Figure 4A and B. C‐Fos expression was enhanced by imipramine but not 9 hrs fasting in CA1, CA2, CA3 and DG of hippocampus compared to control group (CA1: *P *<* *0.05; CA2: *P *<* *0.05; CA3: *P *<* *0.05; DG: *P *<* *0.05). Furthermore, c‐Fos expression was enhanced by DOI only in the CA1 region of hippocampus without affecting other areas of hippocampus compared to fasting control group (*P *<* *0.05).

### BDNF levels in hippocampus and frontal cortex

**Figure 5:**
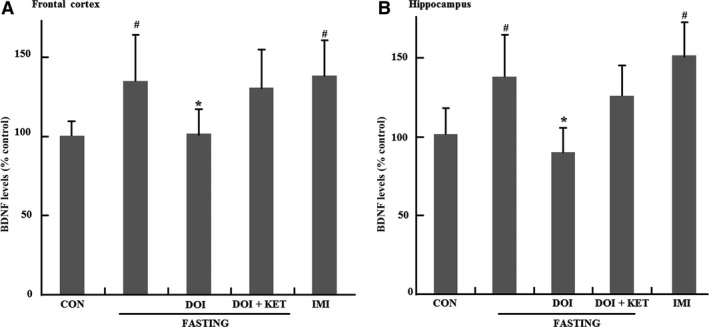
Effects of 9 hrs fasting on BDNF levels, normalize to control, in brain. (**A**) Effects of 9 hrs fasting on BDNF levels in frontal cortex. (**B**) Effects of 9 hrs fasting and DOI on immobility time. Columns represent mean ± S.E.M. (*n* = 7). ^#^ *P *<* *0.05 as compared with control group (non‐fasting); *P <* *0.05 as compared with fasting control group; CON: control; DOI: (±)‐1‐(2, 5‐dimethoxy‐4‐iodophenyl)‐2‐aminopropane hydrochloride, 5 mg/kg, i.p.; IMI: imipramine, 30 mg/kg, i.p.; KET: ketanserin tartrate, 5 mg/kg, i.p.

The effects of 9 hrs fasting on BDNF expression were investigated in frontal cortex and hippocampus in Figure 5. BDNF expression was enhanced by 9 hrs fasting in frontal cortex and hippocampus (Fig. 5A and B) compared to control group (*P *<* *0.05). These enhancements of BDNF levels were inhibited by DOI in both brain regions of fasting mice compared to fasting control group (*P *<* *0.05), and the effect of DOI was also reversed by ketanserin. In addition, BDNF levels were also enhanced in hippocampus and frontal cortex of imipramine‐treated mice (*P *<* *0.05).

## Discussion

In this study, the immobility time was remarkably inhibited by 9 hrs fasting but not 18 hrs in the FST. Furthermore, the antidepressant‐like effects of 9 hrs fasting were inhibited by the 5‐HT_2A/2C_ agonist DOI. These findings were consistent with our studies previously 4. In addition, we have found that the inhibitory effects of DOI on acute fasting‐induced antidepressant effects were also reversed by the 5‐HT_2A_ receptor antagonist ketanserin. This evidence suggests that the antidepressant‐like effects of acute fasting may be exerted *via* 5‐HT_2A_ receptors.

To explore the structural basis on the mechanism of antidepressant‐like effects induced by 9 hrs fasting. In current study, c‐Fos expression was investigated in depression‐related brain regions. C‐Fos expression was increased by 9 hrs fasting in prefrontal cortex (PrL, Cg1 and IL regions) but not hippocampus (CA1, CA2, CA3 and DG) and habenular. A large number of studies have shown that these brain regions (prefrontal cortex, hippocampus and habenular) are suggested to play important roles in depression 6, 11, 21, 22. In this study 9 hrs fasting‐induced c‐Fos expression was significantly further enhanced by DOI in prefrontal cortex (PrL, Cg1 and IL regions), and these effects were also inhibited by ketanserin. Taken together, these data reveal that prefrontal cortex may be the major brain region on the mechanism of the antidepressant‐like effects of acute fasting *via* 5‐HT_2A_ receptors. In our study, imipramine was used as positive control drug. We have found that c‐Fos expression was enhanced by imipramine in hippocampus, frontal cortex and habenular. These data are partially consistent with our previous report that c‐Fos expression was induced by imipramine in the prefrontal cortex 6. These data also indicate that imipramine appears to be more influential on c‐Fos expression in these brain regions compared to acute fasting.

In addition, Pan *et al*. reported that caloric restriction increased BDNF levels in hippocampus and cortex regions, while decreasing BDNF expression in hindbrain and hypothalamic regions that regulate feeding and metabolic efficiency 19. Another finding also showed that BDNF protein levels decreased after 48 hrs fasting and increased after re‐feeding 23. These results suggest that fasting or caloric restriction has various effects on BDNF levels in different brain regions. These interesting data made us further explore whether BDNF expression was also affected by 9 hrs fasting in frontal cortex and hippocampus. In our study, BDNF levels were significantly elevated by 9 hrs fasting in hippocampus and frontal cortex, and increased BDNF levels were also inhibited by DOI. Furthermore, these effects of DOI were also significantly reversed by ketanserin. Previously, we also have reported that 9 hrs fasting also enhanced the ratio of p‐CREB/CREB in the frontal cortex and hippocampus 4. Therefore, these results, combined with our previous data, suggest that the antidepressant‐like effects of acute fasting may be mediated by CREB‐BDNF pathway in frontal cortex and hippocampus circuit.

Fasting can help protect against many brain diseases, such as epilepsy, alzheimer and others. Our findings also suggest that the antidepressant‐like effects of acute fasting may be exerted *via* 5‐HT_2A_ receptor and particularly sensitive to neural activity in the frontal cortex. Furthermore, these antidepressant‐like effects were mediated by CREB–BDNF pathway in frontal cortex and hippocampus. Taken together, these findings suggest that fasting may be potentially helpful against depression.

## Conflict of interest

The authors declare no conflict of interest.
